# Job vacancy at ECDC

**DOI:** 10.2807/1560-7917.ES.2023.28.3.230119v

**Published:** 2023-01-19

**Authors:** Eurosurveillance editorial team

**Affiliations:** 1 European Centre for Disease Prevention and Control (ECDC), Stockholm, Sweden

The European Centre for Disease Prevention and Control (ECDC) invites applications for the following position:

 

- **Communication Specialist**
  The application deadline expires on 9 February 2023.

 

For more information, please visit the ECDC website: 

https://erecruitment.ecdc.europa.eu/?page=advertisement

